# Africa’s Oesophageal Cancer Corridor: Geographic Variations in Incidence Correlate with Certain Micronutrient Deficiencies

**DOI:** 10.1371/journal.pone.0140107

**Published:** 2015-10-08

**Authors:** Torin Schaafsma, Jon Wakefield, Rachel Hanisch, Freddie Bray, Joachim Schüz, Edward J. M. Joy, Michael J. Watts, Valerie McCormack

**Affiliations:** 1 Department of Epidemiology, University of Washington, Seattle, WA, United States of America; 2 Department of Biostatistics, University of Washington, Seattle, WA, United States of America; 3 Section of Environment and Radiation, International Agency for Research on Cancer, Lyon, France; 4 Section of Cancer Surveillance, International Agency for Research on Cancer, Lyon, France; 5 Centre for Environmental Geochemistry, Inorganic Geochemistry, British Geological Survey, Nottingham, United Kingdom; 6 School of Biosciences, University of Nottingham, Sutton Bonington Campus, Loughborough, United Kingdom; Stony Brook University, Graduate Program in Public Health, UNITED STATES

## Abstract

**Background:**

The aetiology of Africa’s easterly-lying corridor of squamous cell oesophageal cancer is poorly understood. Micronutrient deficiencies have been implicated in this cancer in other areas of the world, but their role in Africa is unclear. Without prospective cohorts, timely insights can instead be gained through ecological studies.

**Methods:**

Across Africa we assessed associations between a country’s oesophageal cancer incidence rate and food balance sheet-derived estimates of mean national dietary supplies of 7 nutrients: calcium (Ca), copper (Cu), iron (Fe), iodine (I), magnesium (Mg), selenium (Se) and zinc (Zn). We included 32 countries which had estimates of dietary nutrient supplies and of better-quality GLOBCAN 2012 cancer incidence rates. Bayesian hierarchical Poisson lognormal models were used to estimate incidence rate ratios for oesophageal cancer associated with each nutrient, adjusted for age, gender, energy intake, phytate, smoking and alcohol consumption, as well as their 95% posterior credible intervals (CI). Adult dietary deficiencies were quantified using an estimated average requirements (EAR) cut-point approach.

**Results:**

Adjusted incidence rate ratios for oesophageal cancer associated with a doubling of mean nutrient supply were: for Fe 0.49 (95% CI: 0.29–0.82); Mg 0.58 (0.31–1.08); Se 0.40 (0.18–0.90); and Zn 0.29 (0.11–0.74). There were no associations with Ca, Cu and I. Mean national nutrient supplies exceeded adult EARs for Mg and Fe in most countries. For Se, mean supplies were less than EARs (both sexes) in 7 of the 10 highest oesophageal cancer ranking countries, compared to 23% of remaining countries. For Zn, mean supplies were less than the male EARs in 8 of these 10 highest ranking countries compared to in 36% of other countries.

**Conclusions:**

Ecological associations are consistent with the potential role of Se and/or Zn deficiencies in squamous cell oesophageal cancer in Africa. Individual-level analytical studies are needed to elucidate their causal role in this setting.

## Introduction

Oesophageal cancer (EC) is characterized by a peculiar spatial distribution worldwide, particularly for the histological subtype of oesophageal squamous cell carcinoma (ESCC) which predominates in EC hotspots. Asia’s EC belt has been extensively researched, but little research attention has been given to Africa’s analogous high-incidence area. The latter affects a north-south corridor in easterly lying African countries stretching from Ethiopia and Kenya down to South Africa [1].

Of the environmental factors implicated or postulated to be linked to ESCC [2], in Africa high ethanol-content alcohol and tobacco smoking/chewing contribute in some settings [3;4], but they do not fully explain the burden. Other dominant factors are likely to be co-present to explain the occurrence of this disease in particularly young African patients (under 30 years) and, in some settings, in never-drinkers never-smokers [5;6]. In non-African settings, suboptimal dietary and nutritional intake has been implicated in ESCC. EC risk has been linked to low fruit and vegetable consumption [7] and to diets deficient in certain nutrients, though evidence for the role of individual nutrients is not conclusive. Prospective studies have been few and dietary intervention trials evaluated multiple nutrients, most combinations of which had no effect on EC [8]. Nevertheless, there is some evidence of inverse associations with selenium (Se), including in prospective studies [9], and with zinc (Zn), uniquely from prospective measurements of zinc levels in oesophageal tissue [10;11]. In China, the Linxian general population supplementation trials found that a combination of Se, vitamin-E and beta carotene reduced ESCC mortality if administered under age 55 years [12].

**Fig 1:**
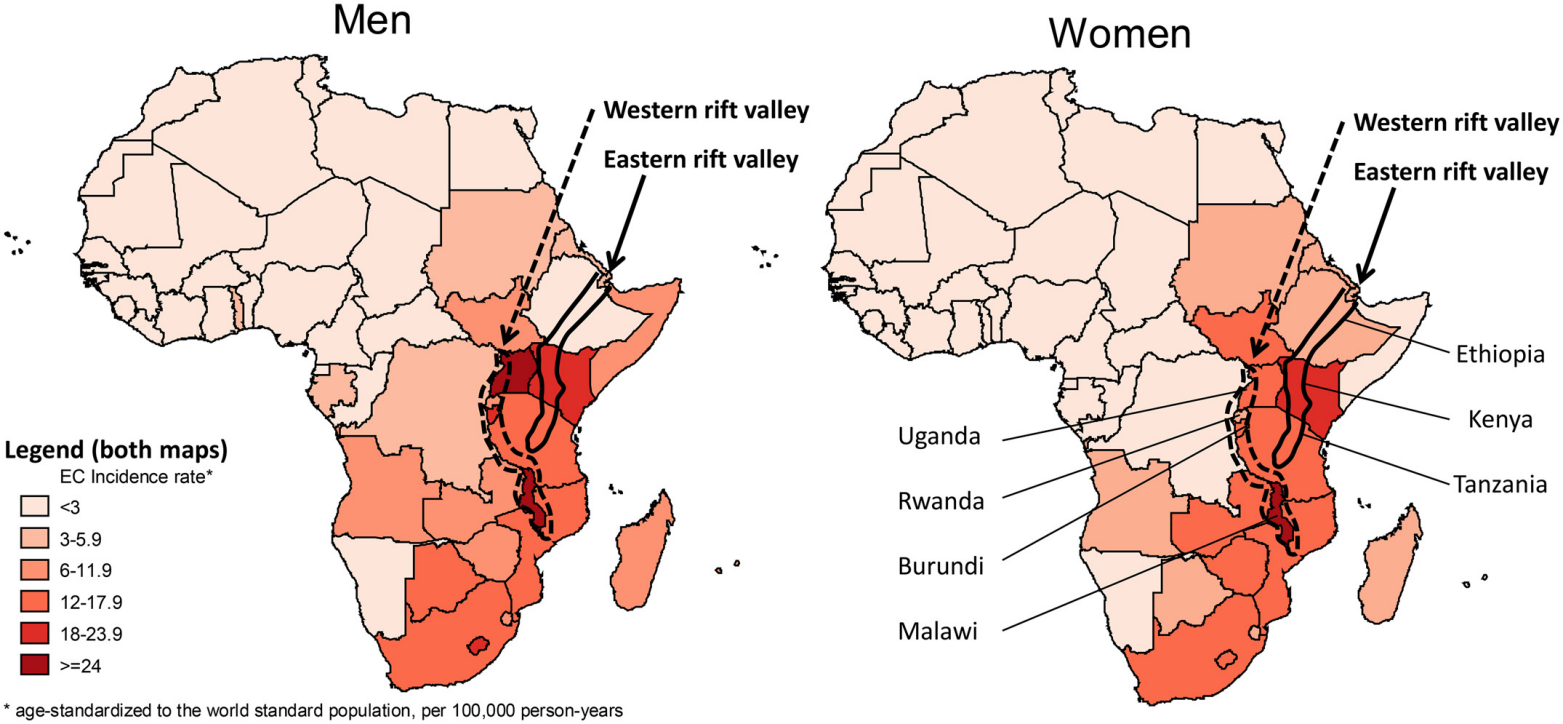
Eastern and western rift valley overlaid on a map of oesophageal cancer incidence rates in Africa, GLOBOCAN 2012. Countries are only named on the graph for women, to simplify the display.

Several primary observations lend plausibility to the potential contribution of nutrition to Africa’s EC corridor. First, restricted cereal-based, mostly maize-based, diets and a reliance on subsistence farming are characteristic of the poorer rural African communities typically affected by the disease [13]. It was been hypothesized that mycotoxin (fumonisin B1) contamination of maize was the underlying carcinogenic agent, but conclusive evidence of its role has not been established. Second, in Africa’s highest EC-incidence country, Malawi, nationwide surveys carried out in 2011 estimated that Se intakes were less than average adult requirements for 80% of the population, primarily due to reduced soil-to-crop Se transfers in the typical low-pH soils [14][15]. Concentrations of biomarkers in the plasma and urine of adults confirmed these observations [15]. Such geochemical factors may be relevant along Africa’s spatially-clustered but culturally-diverse EC corridor. Intriguingly the proximity of this corridor to Africa’s Rift Valley (Fig 1) may be linked to a reliance on locally grown food for basic nutrition, the nutrient levels of which are influenced by local soil geochemistry, thus any excess or deficiencies present are concentrated in a population whose diet predominantly or exclusively originate from this local source. Notably, within the Rift Valley marked in Fig 1 lie all of Africa’s known EC hot spots of Western Kenya (Eldoret, Tenwek), Arsi/Bale regions of Ethiopia, Malawi and Northern Tanzania (Kilimanjaro region). Only South Africa’s high incidence EC area of the Eastern Cape does not fall near this zone. Furthermore, micronutrient deficiencies are common in Africa, particularly for Zn, Se, calcium (Ca) and iodine (I), the prevalence of which has been shown to have large spatial variation [16]. The geospatial correlation of these deficiencies with EC incidence rates has not, to our knowledge, been formally examined in Africa. We do this in the present study, adopting the cautious interpretation necessary for an ecological design. At a country-level we examined gender-specific age-standardized EC incidence rates in relation to dietary supply estimates of seven micronutrients across Africa. These investigations provide valuable preliminary insights into the potential role of micronutrients in the African EC corridor, and they are particularly useful given the lack of long-term cohorts with relevant prospective biomarkers and cancer outcomes.

## Materials and Methods

### Study Design

We performed an ecological analysis to assess the association of country-level dietary micronutrient supplies with gender-specific EC incidence rates across Africa. Potential confounding by two established EC risk factors known to contribute in this region, namely smoking and alcohol intake, were considered.

### Nutrient Supply Data

Estimates of country-level dietary micronutrient supplies in Africa were obtained from recent work by Joy *et al*. which estimated dietary micronutrient supplies and deficiencies at national levels across Africa. In their work, they estimated mean national nutrient supply *per capita* (all ages and both genders combined) by integrating national-level food balance sheets (FBSs), which represent aggregate food supply for 92 edible food items at the retail level and food composition databases. Full details of their methodology are published, and all data extracts included in the present study were obtained from online supplementary tables [16]. In brief, they sourced FBSs for 2009 from the United Nations Food and Agriculture Organization [17]. Nutrient compositions were then assigned to each food item using, depending on the country’s location, one of three regional food composition databases. Those three databases were developed using best-fit estimates from 16 sources. *Per capita* supplies of seven nutrients were estimated overall and by six food sources, for Ca, copper (Cu), iron (Fe), I, magnesium (Mg), Se and Zn. Additionally, we extracted the national estimates of total dietary supplies of energy (kcal) and phytate from that study. Phytate is the principal form of phosphorus storage in plants and is found at high concentrations in cereal and legume seeds. It can chelate with elements including Fe, Mg and Zn, limiting their bioavailability in the human gut.

Using this FBS methodology, dietary nutrient supplies as proxies for nutrient intake were available for 46 African countries (listed in Table 1). National nutrient supplies *per capita* were also compared to nutrient requirements. The estimated average requirement (EAR) of a nutrient is the average daily intake that is estimated to meet the needs of 50% of an age and sex-specific population group. Reference EAR values were used as previously adopted [16;18–20] and in this study we compared nutrient supplies to reference EARs for men aged 30–59 years and women 30–49 years, which are the age bands corresponding to adult-life period likely to be most relevant to the ESCC at-risk period, i.e. prior to disease onset, 80% of which occurs at ages 50+ years in Africa. These references EAR values *per capita per* day in men (M) and women (W) were, for Ca 625 mg (M+W); Cu 700 μg (M+W); Fe 10.5 mg (M)/13.4 mg (W); I 107 μg (M+W); Mg 217 mg (M)/ 183 mg (W); Se 45 μg (M+W); and Zn 11.7 mg (M)/ 8.2 mg (W).

**Table 1:** Summary data by UN sub-region of Africa*: Oesophageal cancer (EC) incidence burden in 2012 (number of cases, age-standardized (world) rate per 100,000 person-years), tobacco and smoking prevalence and mean daily *per capita* nutrient intakes. Countries or territories not included in nutrient supply estimates: Comoros, La Reunion, Mauritius, Somalia, South Sudan, Equatorial Guinea, Western Sahara and Cape Verde.

	African Region	Eastern	Northern	Southern	Middle	Western
	Countries	Ethiopia, Kenya, Malawi, Mozambique, Rwanda, Tanzania, Uganda, Zambia, Zimbabwe, *Burundi* ^†^, *Djibouti* ^†^, *Eritrea* ^†^, *Madagascar* ^†^	Algeria, Egypt, Libya, Morocco, Sudan, Tunisia	Botswana, Namibia, South Africa, Swaziland, *Lesotho^†^*	Cameroon, Congo, Gabon, *DRC* ^†^, *Angola* ^†^, *Central African Rep* ^†^, *Chad* ^†^	Benin, Burkina Faso, Cote D’Ivoire, Gambia, Ghana, Guinea, Mali, Niger, Nigeria, Togo, *Guinea-Bissau* ^†^, *Liberia* ^†^, *Mauritania* ^†^, *Senegal* ^†^, *Sierra Leone* ^†^
No. EC cases	Men	9136	1863	2523	1195	667
	Women	6957	1248	1719	680	343
EC incidence rate	Men	12.0	2.4	13.7	4.2	0.8
	Women	7.9	1.5	6.7	2.0	0.4
Alcohol consumption (liters alcohol capita^−1^ yr^−1^)	Men	8.1	1.9	17.8	8.3	10.5
	Women	2.4	0.2	4.1	2.2	3.2
Tobacco smoking prevalence (%)	Men	15.0	17.7	28.9	29.5	19.6
	Women	10.4	5.0	20.3	23.2	11.2
**Mean daily intake per person** ‡						
Energy (kcal)		2396	3552	2795	2646	2994
Carbohydrate (g)		420	530	423	453	505
Protein (g)		62	110	79	63	73
Fat (g)		43	75	57	53	66
***Micronutrient (symbol*, *units)* EAR** *						
Calcium (Ca, mg)	625	524	613	363	890	702
Copper (Cu, mg)	0.7	1.78	2.67	1.86	1.78	1.85
Iodine (I, μg)	107	151	217	115	146	171
Iron (Fe, mg)	10.5 (M), 13.4 (F)	16.5	22.9	17.7	30.6	36.9
Magnesium (Mg, mg)	217 (M), 183 (F)	462	595	555	679	787
Selenium (Se, μg)	45	36.5	60.2	40.6	55.7	61.7
Zinc (mg)	11.7 (M), 8.2(F)	8.6	16.3	13.2	12.8	14.5
Phytate (mg)	-	2680	2780	2953	2022	2593
Phytate:Zn molar ratio		30.9	16.9	22.2	15.7	17.7

^‡^ Population-weighted

^†^ These 14 countries were excluded hereafter as their incidence estimates are based on neighbouring countries′ data.

*Estimated average requirement (EAR) per capita per day, for adult men (M) age 30–59 and women (F) ages 30–49. If a single EAR is provided, it refers to both sexes. Full details of data sources are provided in the Methods section.

### Cancer Incidence Data

Cancer incidence data were sourced from GLOBOCAN. Country-specific estimates of the number of incident EC cases (ICD–10 C15) in 2012 and corresponding population counts from UN Population Divisions 2012 estimates (United Nations 2013) were obtained from GLOBOCAN, extracted by sex and five-year age group [1]. GLOBOCAN 2012 includes a quality indicator describing the data quality for each country’s incidence estimates. Of the 46 countries with micronutrient data, we excluded 14 countries (indicated in the footnotes to Table 1) for which GLOBOCAN estimates were solely based on rates in neighbouring countries, i.e. no data were available in the country itself (as some countries have no cancer registries). For the countries with estimated new cases of EC in 2012, the estimates refer to all histological subtypes of this cancer, but EC is predominantly ESCC in this region. Indeed, in sub-Saharan Africa age-standardized incidence rates of ESCC are 16 and 20 times higher than the other major histological subtype of oesophageal adenocarcinoma in women and men respectively [21].

### Covariate Information on Confounders

National sex-specific daily tobacco smoking prevalence for adults aged 15+ years for 2012 were obtained from a worldwide study which modelled nationally-representative smoking prevalence data from 1980–2012 [22]. Country-level gender-specific estimates of total alcohol consumption *per capita* at ages 15 years and older in 2008–2010 (total consumption of recorded and unrecorded litres of pure alcohol *per capita per* year) were obtained from WHO [23]. Regional smoking and tobacco prevalence were calculated as averages of country specific prevalence, weighted by the corresponding gender-specific population size.

### Statistical Methods

We plotted age-standardized EC incidence rates (ASR, world standard population, both sexes combined) against daily *per capita* supplies of each micronutrient (log-log scales). We then estimated incidence rate ratios (IRRs) of EC associated with differences in nutrient supplies using a Bayesian hierarchical Poisson-lognormal model [24] for the expected number of EC cases in country *i*: *Y*_*i*_ ~ Poisson(*E*_*i*_ exp[*α* + Σ_j_ *β*_*ij*_ *x*_*ij*_ + *V*_*i*_]), *V*_*i*_|*σ* ^*2*^_*v*_ ~ N(0, *σ* ^*2*^_*v*_), where *σ* ^*-2*^_*v*_ ~ Gamma(0.5, 0.0005) is the prior on the precision of the between-country random effects *V*_*i*_ and *x*_*ij*_ (j = 1,…5) is the value of the *j* ^*th*^ explanatory variable (defined below) in country *i*. Expected numbers of EC cases *E*_*i*,_, separately by sex and 10-year age band (at ages ≥ 20 years), were based on the standard EC incidence rates for the 32 African countries combined multiplied by the corresponding population size. *x*_*1*_ represents log_2_(mean micronutrient), thus 2^β1^ is the IRR associated with a doubling of mean intake; each nutrient was analysed separately. Covariates *x*_*2*_,..,*x*_*5*_ are country-level mean energy and phytate supplies, as well as potential confounders (gender-specific smoking and alcohol). This model accommodates the over-dispersion that is often found when modelling cancer counts, through the random effects. Posterior median estimates of IRRs are reported, along with 95% posterior credible intervals (CIs). Although not all 7 nutrients were hypothesized *a priori* to be related to ESCC (particularly Ca, Cu and I), we analysed associations with each of these nutrients, separately, to ascertain specificity of any observed associations.

## Results

Table 1 provides summary statistics for EC incidence, dietary intakes and potential confounders by region. Estimated ASRs were highest in the Eastern and Southern Africa regions, and in each region they were 1.5 to 2-fold higher in men than in women. Regional differences in alcohol consumption were greatest for men with Southern African men having the highest levels, whereas smoking prevalence was highest in Southern and Middle Africa in both genders (prevalence over 20%). Eastern Africa had the lowest mean intake of macronutrients (overall energy intake, carbohydrates, protein and fat). For micronutrients, Southern Africa had the lowest estimated supplies of Ca and I, and the highest of phytate, whereas East Africa had the lowest mean intakes of Fe and Mg, and average Se and Zn supplies were below EARs.

**Fig 2:**
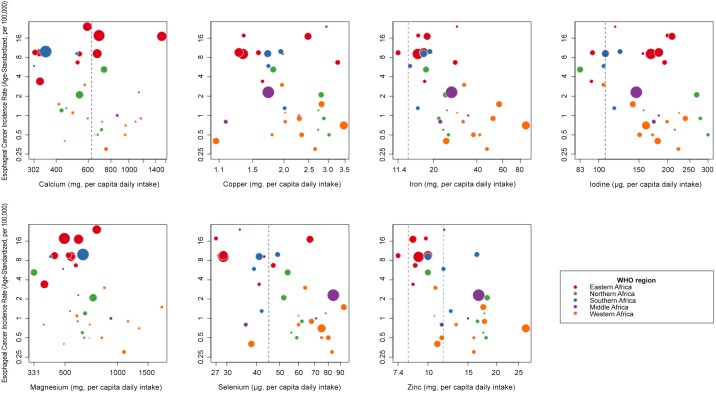
Scatterplots of country-specific oesophageal cancer incidence rates vs mean micronutrient intake per capita. A country’s EC incidence rates are for both sexes combined and are age-standardized to the world standard population. Both axes are plotted on logarithmic scales. Vertical dashed lines indicate estimated average requirements (EAR) in adults: male EARs (blue), female EARs (pink) and non sex-specific EARs (black). For Cu and Mg, and, in men, Fe, EARs lines are suppressed as they are below the mean levels in every country. Circle sizes are proportional to the square root of the total number of EC cases for each country.

Fig 2 shows scatterplots of country-level EC incidence rates (both sexes) *versus* the country’s mean supply of each micronutrient. In each subplot, countries with the highest EC incidence rates at the top are the Eastern (red) and Southern (blue) African countries of Malawi (ASR of 24.2 per 100,000 person-years, with 1969 EC cases), Kenya (ASR 17.6, 3432 cases), Uganda (17.1, 2377 cases), South Africa (ASR 9.9, 3871 cases (large blue point) and Zimbabwe (ASR 9.6, 735 EC cases), and the lowest are the Northern and Western African countries of Nigeria (ASR 0.3, 286 cases), Guinea (ASR 0.4, 24 cases), Gambia (ASR 0.5, 5 cases) and Tunisia (ASR 0.5, 58 cases). Visually, the scatterplots are suggestive of inverse associations for Fe, Mg, Se and Zn. Country-level means were below references EAR for one-third to one-half of countries for Ca, I, and Se and Zn. Few or no countries had nutrient supplies below reference EARs for Cu, Mg and Fe.

**Fig 3:**
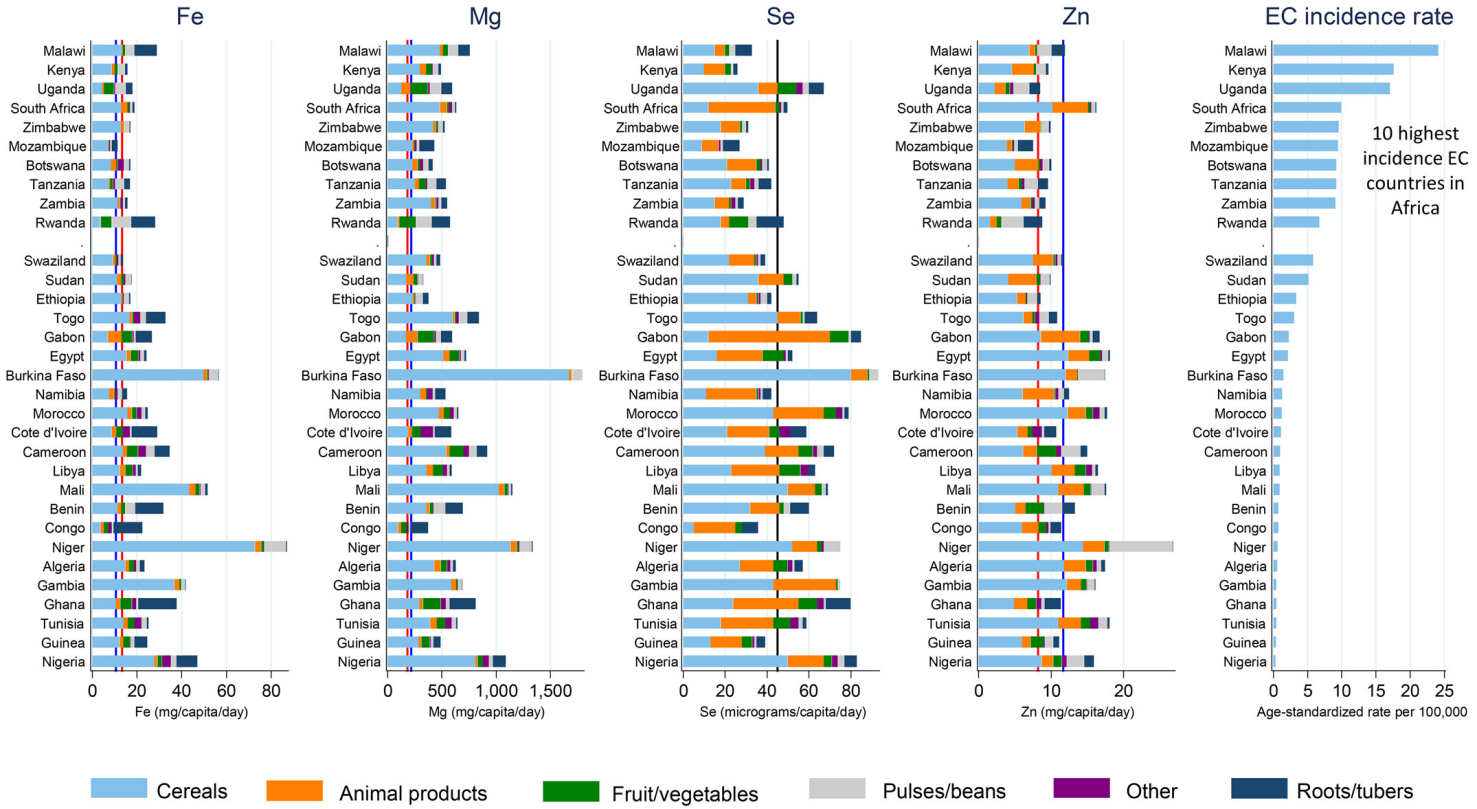
Mean national supplies of Fe, Mg, Se and Zn, overall and by source, Africa, followed by the country’s oesophageal cancer incidence rate. Countries are ranked by esophageal cancer incidence rate (age-standardized, world population, both sexes). Vertical lines indicate the EAR (blue for men, red for women, and black if both sexes are combined.

EC-nutrient associations as estimated by the Poisson regression model are provided in Table 2, by gender and overall (gender-adjusted). Adjusted IRRs for EC associated with a doubling of a country’s mean micronutrient intake indicated null associations for Ca, Cu and I (fully adjusted IRRs 0.74 (95% CI: 0.43, 1.26), 1.11 (0.48, 2.55) and 0.63 (0.30, 1.29) respectively). These null associations held for each gender group and are the 3 nutrients for which there was no a priori hypothesis of an association. For Fe, Mg, Se and Zn, inverse associations were observed and were statistically significant in at least one gender group, with IRRs in both sexes of 0.49 (0.29, 0.82), 0.58 (0.31, 1.08), 0.40 (0.18, 0.90) and 0.29 (0.11, 0.74), respectively. For these latter four nutrients, we examined whether nutrient supply estimates were lower than EARs in the 10 highest EC incidence countries compared to the remaining countries (Fig 3). Nutrient supplies are also displayed according to their sources (Fig 3). For Fe and Mg, national-level supply estimates were not lower than the adult reference EARs. In contrast, for Se and Zn, which were both inversely associated with EC, almost all of the ten highest EC incidence countries had nutrient supplies less than the adult reference EAR, whereas only one-third of the remaining countries had inadequate mean levels. Malawi, Kenya, Zimbabwe and Mozambique were the high-incidence rate EC countries with low mean dietary Se supplies, whereas Mozambique, Kenya, and Uganda were the high EC incidence rate countries with lowest estimated dietary Zn supplies. The sources of these nutrients reveal low supplies from animal products and cereals though they are still the major sources within these populations, and little Zn was sourced from fruit and vegetables in the high-EC incidence rate countries compared to elsewhere (Fig 3).

**Table 2:** Adjusted Incidence rate ratios (IRR) for oesophageal cancer (EC) and their 95% credible intervals (CI) associated with a doubling of a nutrient intake (at a national level), estimated from a Poisson-log normal model.

Gender	Micronutrient	IRR	(95% CI)	IRR	(95% CI)
		Adjusted for age, energy, phytate		Adjusted for age, energy, phytate, alcohol and tobacco	
Male	Calcium	0.66	(0.37, 1.17)	0.82	(0.48, 1.35)
	Copper	0.69	(0.23, 2.11)	0.99	(0.45, 2.19)
	Iron	0.34	(0.21, 0.56)	0.57	(0.34, 0.96)
	Iodine	0.75	(0.33, 1.67)	0.63	(0.31, 1.24)
	Magnesium	0.29	(0.12, 0.68)	0.66	(0.36, 1.21)
	Selenium	0.35	(0.17, 0.73)	0.46	(0.21, 0.97)
	Zinc	0.18	(0.06, 0.53)	0.36	(0.14, 0.91)
Female	Calcium	0.56	(0.24, 1.22)	0.55	(0.22, 1.25)
	Copper	1.09	(0.23, 5.71)	1.42	(0.37, 5.96)
	Iron	0.18	(0.09, 0.33)	0.21	(0.08, 0.45)
	Iodine	0.72	(0.23, 2.20)	0.80	(0.23, 2.64)
	Magnesium	0.09	(0.03, 0.27)	0.27	(0.08, 0.74)
	Selenium	0.24	(0.08, 0.66)	0.18	(0.05, 0.56)
	Zinc	0.06	(0.01, 0.24)	0.10	(0.02, 0.47)
Both sexes	Calcium	0.62	(0.34, 1.09)	0.74	(0.43, 1.26)
	Copper	0.79	(0.25, 2.53)	1.11	(0.48, 2.55)
	Iron	0.30	(0.19, 0.48)	0.49	(0.29, 0.82)
	Iodine	0.71	(0.30, 1.61)	0.63	(0.30, 1.29)
	Magnesium	0.23	(0.10, 0.52)	0.58	(0.31, 1.08)
	Selenium	0.34	(0.16, 0.71)	0.40	(0.18, 0.90)
	Zinc	0.14	(0.05, 0.41)	0.29	(0.11, 0.74)

## Discussion

Building on previous work suggestive of a possible link between nutrient deficiencies and EC risk, we hypothesized that such deficiencies are particularly prevalent in and contribute to the African EC corridor where restricted diets are locally-sourced, and dietary mineral intakes are dependent on the soil mineral content or geochemistry. To investigate this potential link, we performed an ecological study and found that African countries with higher EC incidence rates tended to have lower estimated dietary supplies of Fe, Mg, Zn, and Se. Based on national food supply data, these countries also had greater risks of inadequate dietary supplies of Zn and Se at adult ages, whereas all countries had adequate Mg and Fe supplies. Notably Eastern African countries, where EC rates are highest, had the continent’s lowest estimated supplies of Se and Zn and the highest phytate:Zn molar ratios. In the African EC corridor, maize in the form of maize meal is the main staple and provides 50% of energy intake [16]. Cereals thus provide a large proportion of dietary Se and Zn supplies in EC-affected East Africa, but dietary deficiency remains partly due to low consumption of animal products, especially among lower socioeconomic status and rural populations. On the other hand, cereals are a good source of Mg and Fe, for which no national-level dietary deficiencies were present. No associations were found between EC incidence and I, Cu or Ca. If dietary nutrient deficiencies do play a role, this represents an important modifiable factor in primary prevention efforts for this poor prognosis cancer.

Of specific interest is the finding that the nutrients with both inverse associations with EC and evidence of deficiencies in countries with high incidence rates are the two nutrients for which links to ESCC have been observed in prospective studies outside Africa. For Se, prospective studies in the Netherlands and Linxian, China, found reduced EC incidence rates associated with higher toenail/serum Se levels, with relative risks in the order of 0.80 per standard deviation increase or 0.56 in the highest versus lowest quartiles [9;25]. The Linxian intervention trial has demonstrated a reduction in EC mortality for combined vitamin-E, Se and beta-carotene supplementation if commenced under the age of 55 [12]. For Zn, strong inverse associations are well-established in animals and in humans using local tissue Zn-levels [11], though no reduction in EC incidence was found using a multivitamin supplement that included Zn in the above-mentioned trial [12]. Their protective mechanisms may be as anti-oxidants, in tissue and cell-repair pathways in Se-containing glutathione enzymes, mucosal growth and maintenance (for Se and vitamin-E), and, for Zn, in counteracting the effect of certain nitrosamines [26]. If dietary nutrient deficiencies do play a role, the manifestation of cancer particularly at this anatomical site might be due to nutrient deficiencies rendering individuals more susceptible in the presence of direct genotoxic carcinogens to the oesophageal mucosa. Agents such as potent alcohols, tobacco, thermal injuries or polycyclic-aromatic hydrocarbon exposures (e.g. in household air pollution) are known to be prevalent in at least some parts of the African EC corridor [27–31], but a full evaluation of these environmental factors and their interactions with nutrients on EC risks has not been conducted.

The present investigation is exploratory and its results must be considered in light of limitations of its design, as well as the respective estimation methodologies of both the exposure and outcome data. For cancer data, in an attempt to overcome uncertainties in incidence rates, we excluded countries with the lowest estimate quality index, as assessed by GLOBOCAN. However the included incidence rates are subject to differential rates of diagnosis and to completeness of registries, especially in countries lacking population-based cancer registries [32]. Nevertheless, the order of magnitude (over ten-fold) of between-country differences in EC incidence rates indicates plausibility in the broad ranking of countries; and for the highest EC incidence countries, published reports of counted diagnosed cases can be found [27;30;33–37]. Further, case series of pathological findings from upper gastrointestinal endoscopy patients are also consistent with such large differences—e.g. in 1034 such patients in Malawi, 27% had EC [38], whereas in a series of 3110 patients in the Ghana, West Africa, only one patient had EC (located in the distal oesophagus) [39]. In terms of nutrient supply estimates, national-level FBSs provide a proxy for food supply and may over-estimate supply by not accounting for household-level food waste or under-estimate supply by not capturing some subsistence production. To control for any potential over or under-estimation at a national level, we adjusted for total energy supply, In addition, dietary nutrient supply estimates depend on the accuracy and relevance of food composition data. However, the method remains useful in populations for which neither surveys of dietary intake nor biomarker-based nutritional status have been conducted in nationally-representative populations. [14].

The ecological design gives rise to potential ecological bias, which may be worsened due to the expansive geographical areas of African countries. If food supply data were available at district/province-levels this would enable a higher granularity of data and scatterplot points might align differently. The need for a smaller-scale of analysis is greater because the influence of the local physical environment on human health is likely to be greater in settings where there is a reliance on a narrow range of locally-sourced foods. For example, fish is a good source of Se, thus intakes in populations residing along Africa’s large coastal and lake areas may differ to those residing far from major water bodies. Greater spatial resolution of food composition data would also improve the study. Despite the large-scale of analysis, studies of sub-populations in East Africa, including analyses of weighed duplicate diets and biomarkers, are consistent with the observation of a high prevalence of dietary Se and Zn deficiencies in high EC incidence regions. The plant uptake of Se from soils is under strong geochemical control and studies in Malawi have demonstrated that the concentration of Se in maize grain and other crops, and thus dietary Se supply and nutritional status, varies greatly between populations living on different soil types [15;40;41]. As well as Se deficiencies, a high prevalence of Zn deficiencies has also been noted in Malawi, validating the FBS-based estimates [42]. Additionally in South Africa, geographical patterns of soil pH and low concentrations of Se in maize collected from silos have also been documented [43]. Additionally, previous work showed that populations living in the historically high EC incidence area in the Eastern Cape province of South Africa had low whole blood concentrations of Se but not Cu/Zn/Mg levels [44]. As explained in the introduction, because of the very unusual north-south eastern corridor of EC in Africa, EC susceptibility in this setting may well be driven by an external environmental factor, such as diet and its geochemical influences. Many of the African EC hotspots are located along the Rift Valley, yet there is no obvious lifestyle factor known to explain this distribution that spans geographically connected areas but affects culturally and genetically diverse populations, e.g. the Kalenjin people in West Kenya and women in the Ethiopian highlands.

We now discuss statistical considerations. In general, ecological regression studies such as that reported here are potentially susceptible to confounding by location, whereby unmeasured area-level covariates may be at least partially responsible for the observed associations with exposures of interest. Unfortunately, no reliable method is available to control for such confounding. One approach is to include spatial random effects in the model to account for residual spatial correlation between rates according to the geographical positioning of countries. However such spatial effects can attenuate associations by diluting the exposure signal [24], and so we chose to not pursue this option. The positive correlations between nutrients prohibited the possibility of examining their independent associations and/or nutrient-nutrient interactions (correlation coefficients>0.64 for Mg, Fe, Se and Zn). Further, dietary supply estimates are not gender specific, thus associations with gender-specific EC incidence rates add limited additional information.

Given the suggestive findings, but limitations of the study design, further investigation in individual-level analytic studies is warranted in Africa where large mature prospective studies are lacking. The design of individual-level analytical studies that include a nutrition element need careful consideration, as biomarkers of short-term exposures taken from dysphagia-suffering EC patients may be poorly representative of long-term exposures. Attempts to use longer-term biomarkers in toenails have not been rewarding, owing to contamination by embedded soil. If validated however, exposures measured in household members might be useful as surrogates of patients’ status. Furthermore, if the etiologic factors are ubiquitous, matching on residential location or setting, a study in a locally-focused area might inadvertently reduce exposure heterogeneity by matching on the causal environmental factors that are the drivers of the geographical patterns. Conducting research in areas that span geographically-proximate areas with sharp gradients in EC incidence rates may be more insightful for this purpose, as have been conducted in the Eastern African region for other cancers, e.g. to investigate micro-geographical variations in Burkitt’s lymphoma incidence [45]. Finally, ESCC is multifactorial and if nutrition plays a role in Africa, it is likely to act by generating a population susceptible to other direct genotoxic agents. Factors with putative contributions to the EC African corridor that need further investigation include alcohol, tobacco, polycyclic aromatic hydrocarbons, acetaldehyde, hot beverages, N-nitrosamines, infections and mycotoxins [2]. The latter is of particular relevance here, as higher EC incidence rates associated with maize based diets have been suggested, largely based on ecological studies, to be due to fumonisin derivatives sourced from contaminated maize. Individual level studies are thus needed to illuminate whether associations are causal and what pathways are involved.

Africa experiences a heavy burden of EC (27,500 new cases and 25,000 deaths in 2012) [1]. If micronutrient deficiencies contribute to this burden, the potential preventive possibilities are real, through dietary diversification and increased consumption of animal-based or other rich sources of Se and Zn. Some commercially milled super maize meals sold in South Africa are already fortified with these and other nutrients (e.g. with zinc oxide, B_12_, Se as per package labels on maize meal available in supermarkets). Agronomic biofortification of staple crops is another possibility, as carried out nationwide for Se in Finland and New Zealand since the 1980s. Studies have already demonstrated that this is possible on low-pH soils in Africa using selenate-containing fertilizers to increase maize-Se levels [46]. The combination of the plausibility of micronutrients in EC aetiology as well as the potential for prevention warrant further in-depth individual level research in countries along the African EC corridor.

## Supporting Information

S1 STROBE Checklist (DOC)
