# Correction: Africa's Oesophageal Cancer Corridor: Geographic Variations in Incidence Correlate with Certain Micronutrient Deficiencies

**DOI:** 10.1371/journal.pone.0142648

**Published:** 2015-11-13

**Authors:** Torin Schaafsma, Jon Wakefield, Rachel Hanisch, Freddie Bray, Joachim Schüz, Edward J. M. Joy, Michael J. Watts, Valerie McCormack

Fig 2 is incorrect. The authors have provided a corrected version here.

**Fig 2:**
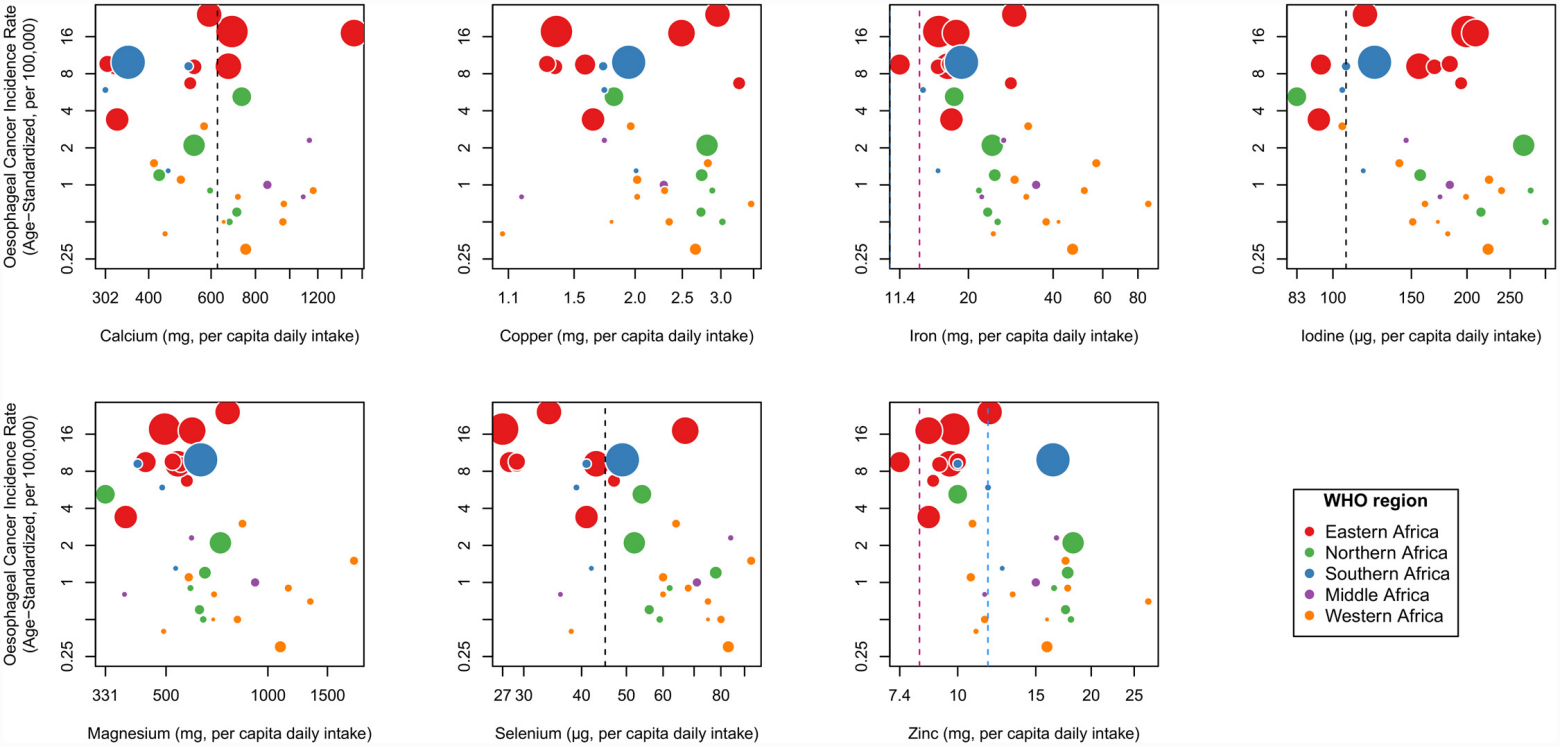
Scatterplots of country-specific oesophageal cancer incidence rates vs mean micronutrient intake per capita. A country’s EC incidence rates are for both sexes combined and are age-standardized to the world standard population. Both axes are plotted on logarithmic scales. Vertical dashed lines indicate estimated average requirements (EAR) in adults: male EARs (blue), female EARs (pink) and non sex-specific EARs (black). For Cu and Mg, and, in men, Fe, EARs lines are suppressed as they are below the mean levels in every country. Circle sizes are proportional to the square root of the total number of EC cases for each country.
