# Supplementary material for: Africa’s Oesophageal Cancer Corridor: Geographic Variations in Incidence Correlate with Certain Micronutrient Deficiencies
Source: PLoS One. 2015 Oct 8;10(10):e0140107. doi: 10.1371/journal.pone.0140107 (PMC4598094; doi:10.1371/journal.pone.0140107)
Supplement: S1 STROBE Checklist — (DOC) [file pone.0140107.s001.doc]

**S1 Stroke Checklist**

**Supplementary Information**

STROBE Statement—Checklist of items that should be included in reports of ***cross-sectional studies***

*Author’s insertions and responses to each checklist item are provided in underlined italics.*

*The study is an ecological analysis, whose design is most closely related to a cross-sectional study, being a study cross-sectional in time but with aggregate level exposure and outcome data.*

|  | Item No | Recommendation |
| --- | --- | --- |
| **Title and abstract** | 1 | Indicate the study’s design with a commonly used term in the title or the abstract.  *Ecological study – abstract line 5.* |
|  |  | (*b*) Provide in the abstract an informative and balanced summary of what was done and what was found. *Core method provided and main results appear in the abstract.* |
| Introduction | | |
| Background/rationale | 2 | Explain the scientific background and rationale for the investigation being reported.  *This is in the ‘Background’ section of the abstract.* |
| Objectives | 3 | State specific objectives, including any prespecified hypotheses.  *Page 4 lines 67-72.* |
| Methods | | |
| Study design | 4 | Present key elements of study design early in the paper.*These are provided first on page 4 line 68 and again at the start of the Materials and Methods section (‘Study Design’)* |
| Setting | 5 | Describe the setting, locations, and relevant dates, including periods of recruitment, exposure, follow-up, and data collection.  *Table 1 indicates the countries included in analyses, and also notes the countries that could not be included due to lower quality cancer incidence data.* |
| Participants | 6 | (*a*) Give the eligibility criteria, and the sources and methods of selection of participants. *Eligibility of countries in the analysis are explained in the materials and methods sections, lines* |
| Variables | 7 | Clearly define all outcomes, exposures, predictors, potential confounders, and effect modifiers. Give diagnostic criteria, if applicable.  *These are provided in headed sections of the materials and methods.* |
| Data sources/ measurement | 8* | For each variable of interest, give sources of data and details of methods of assessment (measurement). Describe comparability of assessment methods if there is more than one group.  *In the Materials and Methods section, data sources for each of the outcome (Cancer data), exposures of interest (nutrient supply data) and confounders are provided.* |
| Bias | 9 | Describe any efforts to address potential sources of bias  *Ecological bias and its potential extent is included in the Discussion, lines 254-275.*  *Statistical considerations are discussed in lines 276-285.* |
| Study size | 10 | Explain how the study size was arrived at.  *All eligible Africa countries with data were included, as explain in the Materials and Methods section.* |
| Quantitative variables | 11 | Explain how quantitative variables were handled in the analyses. If applicable, describe which groupings were chosen and why.  *Statistical Methods lines 139-149* |
| Statistical methods | 12 | (*a*) Describe all statistical methods, including those used to control for confounding |
|  |  | (*b*) Describe any methods used to examine subgroups and interactions  *Gender specific results are also provided.* |
|  |  | (*c*) Explain how missing data were addressed.  *There were no missing, as per inclusion criteria in the analyses.* |
|  |  | (*d*) If applicable, describe analytical methods taking account of sampling strategy.  *Not applicable. All eligible countries were included.* |
|  |  | (*e*) Describe any sensitivity analyses.  *Not conducted.* |
| Results | | |
| Participants | 13* | (a) Report numbers of individuals at each stage of study—eg numbers potentially eligible, examined for eligibility, confirmed eligible, included in the study, completing follow-up, and analysed. |
|  |  | (b) Give reasons for non-participation at each stage. *Excluded countries are listed in table 1.* |
|  |  | (c) Consider use of a flow diagram. *We did not include a flow diagram, as the inclusion of countries is a simple calculation with just two substractions for no food balance sheets and the lowest quality cancer incidence data.* |
| Descriptive data | 14* | (a) Give characteristics of study participants (eg demographic, clinical, social) and information on exposures and potential confounders |
|  |  | (b) Indicate number of participants with missing data for each variable of interest |
| Outcome data | 15* | Report numbers of outcome events or summary measures |
| Main results | 16 | (*a*) Give unadjusted estimates and, if applicable, confounder-adjusted estimates and their precision (eg, 95% confidence interval). Make clear which confounders were adjusted for and why they were included. *These are provided in Table 2.* |
|  |  | (*b*) Report category boundaries when continuous variables were categorized. *Not applicable.* |
|  |  | (*c*) If relevant, consider translating estimates of relative risk into absolute risk for a meaningful time period. *As this is not individual level data, we cannot do this.* |
| Other analyses | 17 | Report other analyses done—eg analyses of subgroups and interactions, and sensitivity analyses. *Gender specific results are discussed.* |
| Discussion | | |
| Key results | 18 | Summarise key results with reference to study objectives -  *first paragraph of the discussion.* |
| Limitations | 19 | Discuss limitations of the study, taking into account sources of potential bias or imprecision. Discuss both direction and magnitude of any potential bias. *Limitations of the study are discussed on lines 244 to 285.* |
| Interpretation | 20 | Give a cautious overall interpretation of results considering objectives, limitations, multiplicity of analyses, results from similar studies, and other relevant evidence.  *Because this is an ecological design, we reiterate the very exploratory nature of the findings in the abstract, introduction and again in the discussion. Nevertheless the observations are interesting and they highlighted much needed research avenues worthy of pursuit.* |
| Generalisability | 21 | Discuss the generalisability (external validity) of the study results. *The results are discussed in relation to Africa.* |
| Other information | | |
| Funding | 22 | Give the source of funding and the role of the funders for the present study and, if applicable, for the original study on which the present article is based.  *There was no external specific funding for this work.* |

*Give information separately for exposed and unexposed groups.

**Note:** An Explanation and Elaboration article discusses each checklist item and gives methodological background and published examples of transparent reporting. The STROBE checklist is best used in conjunction with this article (freely available on the Web sites of PLoS Medicine at http://www.plosmedicine.org/, Annals of Internal Medicine at http://www.annals.org/, and Epidemiology at http://www.epidem.com/). Information on the STROBE Initiative is available at www.strobe-statement.org.
